# A Nonsense Mutation in the Acid α-Glucosidase Gene Causes Pompe Disease in Finnish and Swedish Lapphunds

**DOI:** 10.1371/journal.pone.0056825

**Published:** 2013-02-14

**Authors:** Eija H. Seppälä, Arnold J. J. Reuser, Hannes Lohi

**Affiliations:** 1 Research Programs Unit, Molecular Medicine, University of Helsinki, Helsinki, Finland; 2 Department of Veterinary Biosciences and Department of Medical Genetics, University of Helsinki, Helsinki, Finland; 3 Folkhälsan Institute of Genetics, Helsinki, Finland; 4 Departments of Clinical Genetics and Paediatrics, Center for Lysosomal and Metabolic Diseases, Erasmus MC, Rotterdam, The Netherlands; University Hospital S. Maria della Misericordia, Udine, Italy

## Abstract

Pompe disease is a recessively inherited and often fatal disorder caused by the deficiency of acid α-glucosidase, an enzyme encoded by the *GAA* gene and needed to break down glycogen in lysosomes. This glycogen storage disease type II has been reported also in Swedish Lapphund dogs. Here we describe the genetic defect in canine Pompe disease and show that three related breeds from Scandinavia carry the same mutation. The affected dogs are homozygous for the *GAA* c.2237G>A mutation leading to a premature stop codon at amino acid position 746. The corresponding mutation has previously been reported in humans and causes infantile Pompe disease in combination with a second fully deleterious mutation. The affected dogs from both the Finnish as well as the Swedish breed mimic infantile-onset Pompe disease genetically, but also clinico-pathologically. Therefore this canine model provides a valuable tool for preclinical studies aimed at the development of gene therapy in Pompe disease.

## Introduction

Pompe disease, also referred to as glycogen storage disease type II, is an autosomal recessive lysosomal storage disorder caused by a deficiency of acid α-glucosidase (EC 3.2.1.20) in humans. The enzyme degrades α-1,4 and α -1,6 linkages in glycogen, maltose, and isomaltose. Deficiency of this enzyme leads to storage of glycogen inside lysosomes and eventually also to cytoplasmic glycogen storage. Ultimately, the accumulation of glycogen results in tissue destruction reflected by a spectrum of clinical phenotypes ranging from a fatal infantile form of Pompe disease to a slowly progressive late-onset form [1], [2].

Canine glycogen storage disease type II was first described in a Lapland dog from Sweden by Mostafa in 1970 [3]. In the 1980s researchers from the Netherlands reported clinical and morphological data of another Swedish Lapphund family affected by the same disease [4], [5]. Clinical signs and symptoms of the affected dogs included oesophageal dilation induced vomiting, progressive muscular weakness, loss of condition, clinical heart disease, and myocardial hypertrophy, the severity of which required euthanasia at about 1.5 years of age [3], [4], [6], [7]. Pathological findings revealed accumulation of glycogen containing vacuoles in cerebral cortex, liver, and myocardial plus oesophageal smooth muscle, and ultrastructural evidence of a lysosomal glycogen storage disorder [8]. Biochemical studies demonstrated a severe deficiency of acid α-glucosidase activity in heart, skeletal muscle and liver [9]. Furthermore, it was shown that the presence of catalytically inactive acid α-glucosidase was the primary defect in canine Pompe disease and, consequently, that Swedish Lapphunds represented a homologous model of human classic infantile-Pompe disease [5], [6]. However, the primary molecular genetic defect of canine Pompe disease has remained unknown for more than 40 years.

In the present study we show that a recessively inherited nonsense mutation in the acid α-glucosidase (*GAA*) gene causes Pompe disease in both Swedish and Finnish Lapphunds as it does in humans. In addition, we discovered that the same mutation occurs in Lapponian Herders although no cases in that breed have been reported as yet. Forty years after the initial cases were reported our findings have finally enabled developing a genetic test to eradicate lethal Pompe disease from the Scandinavian dog breeds. Our study has also established affected Swedish and Finnish Lapphunds as large animal models for the preclinical testing of gene therapy in Pompe disease.

## Materials and Methods

### Study population

Four out of seven puppies from a Finnish Lapphund litter born in 2010 were affected by slowly progressive muscle weakness and from frequent vomiting. The affected dogs show a striking mutual resemblance indicating a genetic disease and when clinical history and findings were compared to the previous literature describing inherited diseases in Lapland dogs, canine Pompe disease was strongly suspected [3], [4], [6]. We collected blood samples from two affected dogs. Case 1 suffered from regurgitation starting at the age of 7 months. At the age of 8 months the owner started to notice progressive muscle weakness and at the age of 12 months the dog started to vomit mucous. Constant panting, dysphonia and apathetic habitus were evident at the age of 13 months. The local veterinarian had taken radiographs of heart (age 15 months) and thorax (age 18 months) and had performed abdominal ultrasound examination (age 15 months) for case 1. Radiographs showed dilatation of the esophagus and cardiac enlargement. Ultrasound examination revealed changes in the liver. Blood chemistry showed decreased plasma alkaline phosphatase (ALP) level and increased alanine aminotransferase (GPT, ALAT), blood urea nitrogen (BUN) and glucose levels and hematology results showed increased monocyte count and increased mean platelet volume at the age of 18 months. Case 1 is currently alive at two years of age but is not expected to survive for very much longer.

**Figure 1:**
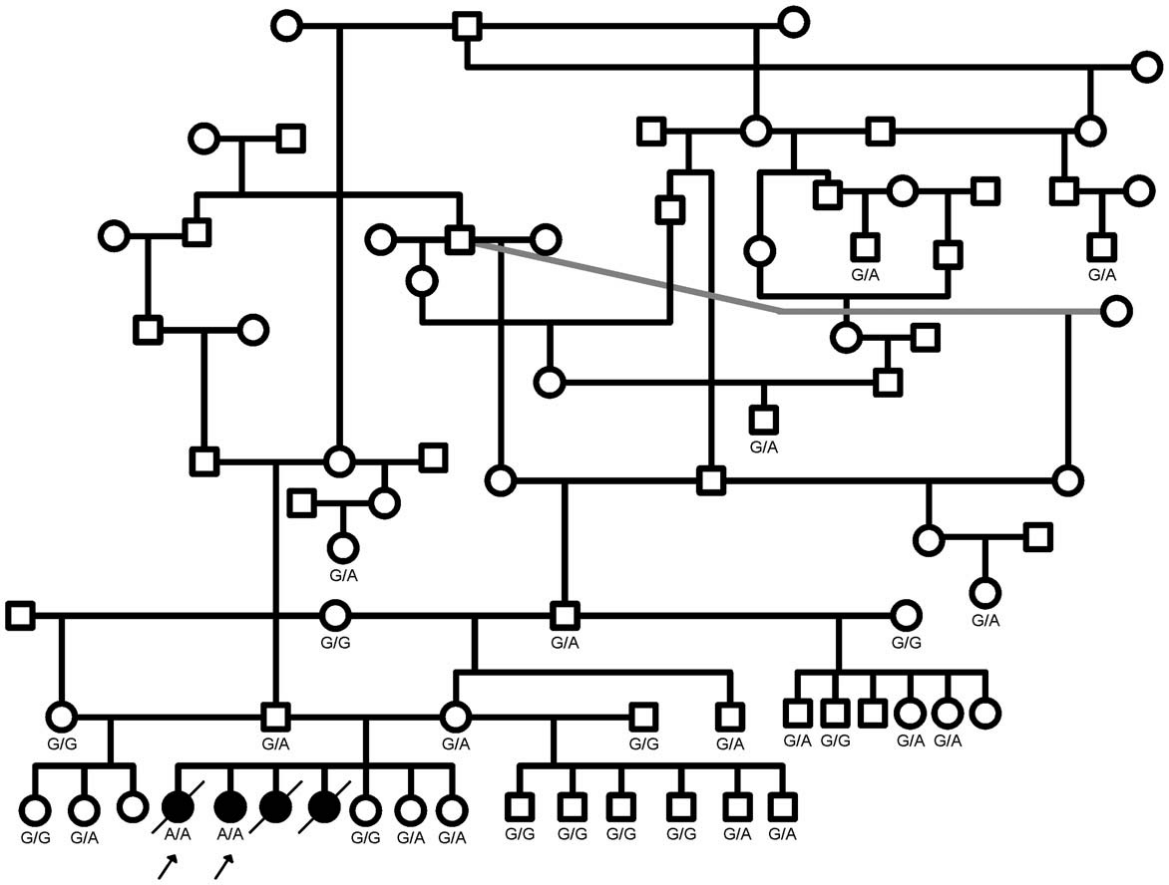
The pedigree of the Finnish Lapphunds affected with Pompe disease. Four puppies suffered similar symptoms and two of them (arrows) were available for genetic studies. All five mutation carriers that were identified by screening of 95 healthy Finnish Lapphunds could be traced back to the same pedigree. The *GAA* c.2237G>A genotypes of the studied dogs are shown below the gender symbol.

The owner of case 2 reported that the dog gradually developed symptoms and loss of condition at the age of 12 months. The coat became poor quality, appetite was lost and dog was vomiting. As the symptoms progressed the local veterinarian examined the dog first time when she was 14 months old. Blood chemistry showed increased plasma ALAT and aspartate aminotransferase (GOT, ASAT) levels at the age of 15 months and they had increased further at the age of 18 months and the dog was euthanized at the age of 19 months. The common symptoms for both of these siblings were vomiting of mucous, panting, dyspnea, dysphagia, regurgitation and progressive muscle weakness. Blood was drawn also from three healthy litter mates, both parents and 18 healthy second degree relatives (Fig 1).

We also analyzed a DNA sample from a previously described Swedish Lapphund with Pompe disease born in 1979 (case four reported in [7]). This case was diagnosed at 4 months of age by measuring low acid α-glucosidase activity in peripheral blood leucocytes. The clinical symptoms started to appear at the age of 7 months and the dog was euthanized at the age of 14 months when clinical disease developed.

### Mutation detection and screening

The coding exons and splice junctions of the canine *GAA* gene were amplified by PCR. The analysis included DNA from the two affected Finnish Lapphunds, their dam and an unrelated healthy 8-year-old Finnish Lapphund as control. The PCR products were purified with ExoSAP-IT kit (USB Corporation, Cleveland, Ohio) before sequencing with an ABI Prism 3730xl DNA analyzer (Applied Biosystems, Foster City, CA). Sequence data was analyzed by Sequencher® 5.0 software, (Gene Codes Corporation, Ann Arbor, MI USA). The primers are available upon request.

To determine the population frequency of the newly identified mutation we screened 95 Finnish Lapphunds, 99 Lapponian Herders and 34 Swedish Lapphunds. Most of the Finnish Lapphunds and Lapponian Herders were unrelated at the grandparental level, whereas many of the Swedish Lapphunds were related to each other. A total of 304 samples from 21 other breeds were also screened for the mutation (Table 1).

**Table 1:** Other breeds screened for the *GAA* p.W746* mutation.

Breed	Number of samples tested
Alaskan Malamute	17
Basenji	15
Bernise Mountain Dog	16
Bichon Havanaise	16
Brasilian Terrier	14
Curly Coated Retriever	13
Dachshund, wire-haired	14
Finnish Hound	16
Finnish Spitz	21
German Shepherd	14
Irish Setter	14
Keeshond (Wolfsspitz)	15
Miniature Pincher	13
Rough Collie	11
Saluki	18
Samoyed	13
Schipperke	14
Siperian Husky	11
Spanish Water Dog	15
Staffordshire Bull Terrier	9
Whippet	15
TOTAL 21 BREEDS	304

None of the tested dogs carried the mutation.

### Analysis of *GAA* mRNA

Blood samples from one affected (homozygous mutant) and one unaffected (homozygous wild type) Finnish Lapphunds were collected to PAXgene Blood RNA Tubes and total RNA was purified using RNeasy Mini Kit (Qiagen AB, Scandinavia, Solna, Sweden). Then mRNA was reverse transcribed to cDNA using High Capacity RNA-to-cDNA Kit (Applied Biosystems, Foster City, CA). Part of the *GAA* cDNA was sequenced using exon-specific primers (available upon request).

### Ethics statement

All samples were from privately owned pets and we obtained consent from the owners of the dogs involved in our study. We also obtained permission for blood sampling from the Animal Ethics Committee (ESLH-2009-07827/Ym-23), The State Provincial Office of Southern Finland, P.O. B150, 13101 Hämeenlinna.

## Results

As part of our canine disease genomics program, we became interested in identifying the molecular basis of Pompe disease in dogs when we encountered a Finnish Lapphund litter with four affected dogs. Their symptoms and their clinical findings were consistent with those previously described in four Swedish Lapphunds with Pompe disease [3], [4], [7]. As a first action, we collected blood samples from two of the four affected Finnish dogs, from three of their unaffected littermates and from their parents for the purpose of DNA extraction.

Exonic sequencing of the canine *GAA* gene revealed a c.2237G>A change, compared to the reference sequence RefSeq XM_845556.2, that results in a premature stop codon p.W746* in the 15^th^ coding exon. The nucleotide numbering reflects the cDNA numbering with +1 corresponding to the A of the ATG initiation codon; the amino acid numbering is according to XP_850649.1. The p.W746* mutation truncates the last 206 amino acids (∼22% of the protein) of the canine acid α-glucosidase and its effect is very severe according to the Pompe Disease Mutation Database at www.pompecenter.nl. The two affected Finnish Lapphund littermates were both homozygous for the mutation, their parents were both heterozygous, two of their three healthy littermates were heterozygous and one was homozygous wild type (Fig. 1). In addition, ten synonymous coding variants and seven intronic variants were detected in *GAA* gene but none of them segregated with the disease.

It then became interesting to investigate if the Swedish Lapphunds that had been described back in the 1960s and 1970s, when DNA analysis was not yet possible, had the same or another Pompe disease mutation. Fortunately, a cell line established in those days from the tong tissue was successfully retrieved from storage in liquid nitrogen and could serve as a DNA source. Thereby, we were able to establish that one of the originally described Swedish Lapphunds with Pompe disease was also homozygous for the same c.2237G>A mutation.

We then aimed to screen the current Finnish and Swedish Lapphund populations, the Lapponian Herders and 22 other breeds for the occurrence of the c.2237G>A mutation. This study revealed that the mutation is present only in two Scandinavian breeds: 5% (5/95) of Finnish Lapphunds and 2% (2/99) of Lapponian Herders carry the mutation. The cohort of 34 Swedish Lapphunds did not contain any carriers.

Sequencing of the GAA mRNA from homozygous mutant Pompe case and from wild type healthy control revealed that the mutated gene is transcribed into mRNA suggesting that it does not lead to large-scale nonsense-mediated RNA decay (data not shown). This is consistent with the previous study showing that the mutated GAA protein is also present in the affected dogs, although in an inactive form [6].

## Discussion

Pompe disease is an autosomal recessive disorder in humans caused by mutations in the *GAA* gene (OMIM #232300, [10], [11]). Acid α-glucosidase is encoded by the *GAA* gene. It is essential for the degradation of glygogen to glucose in lysosomes. Over 300 potentially pathogenic *GAA* mutations have been identified in human patients (www.pompecenter.nl). Human patients are often compound heterozygotes. A combination of two mutated alleles deleting the enzymatic function completely results in infantile-onset Pompe disease. Different combinations of other mutations in the *GAA* gene affect GAA activity and modify the onset and progression of the disease. However, at present, it is not possible to predict in all cases the clinical course based on genotype alone [12].

Besides humans, Pompe disease has also been reported in other species including cattle (OMIA 000419-9913, [13], [14]), Japanese quails (OMIA 000419-93934, [15]), cats (OMIA 000419-9685, [16]), sheep (OMIA 000419-9940,[17]) and dogs (OMIA 000419-9615, [3], [4]). Three breed-specific mutations in bovine *GAA* gene all causing a premature stop codon have been identified in Brahman and Beef Shorthorn cattle [18]. So far no mutations have been identified in cats, quails or sheep with Pompe disease. Until now, the genetic defect underlying canine Pompe disease has also remained unknown.

The clinical, pathological and biochemical characteristics of classic infantile Pompe disease in humans and dogs resemble each other very well [6]–[8]. The pathogenesis in both species implies the total absence of acid α-glucosidase activity, generalized glycogen storage leading to progressive muscular weakness, cardiac hypertrophy, cardio-respiratory failure and death. A finding unique to the disease in dogs is the megaoesophagus and its associated symptoms of regurgitation and vomiting [8]. With respect to this manifestation, the difference between humans and dogs might be related to their different posture. Swallowing difficulties are amongst the clinical manifestations of Pompe disease in humans, but are neither associated with dilatation of the oesophagus nor do they lead to vomiting.

The p.W746* nonsense mutation was first reported in combination with a second fully deleterious mutation (c.2758_2775dup) in a patient with infantile onset Pompe disease [19]. Later, four Italian infants were reported to carry this mutation in four different heteroallelic combinations with either c.525delT, c.670C>T, c.1655T>C, or c.2481+102_2646+31del [20]–[22]. In addition, 9/40 Italian patients with late onset forms of Pompe patients were identified as carriers of the p.W746* mutation. All of them had the most common leaky splice variant c.-32-13T>G as second mutation [23]. W746* has also been reported in heteroallelic combination with the less severe P285S variant and was in that case associated with late-onset disease [24]. The fact that W746* was rather frequently seen in humans and is now also found in dogs suggests that the chromosomal region around c.2237G is a mutation hotspot. The listing of c.2236T>G, c.2237G>C, c.2238G>C, c.2238G>A as other pathogenic mutations in human Pompe disease supports this view (www.pompecenter.nl).

Although the affected Swedish Lapphund born in 1979 was homozygous for the mutation and original reports indicate that there have been several cases among Swedish Lapphunds in those days [3]–[7], we were not able to identify any carriers from the current dogs of this breed. This might be due to too small sample size. It is also possible that the rigorous breeding program has eliminated the carriers from the modern Swedish Lapphunds. It is also worth noting that we identified carriers from Lapponian Herders although no affected cases have been reported in this breed.

The canine p.W746* mutation is shared exclusively among the three closely related breeds from Lapland reflecting a founder effect. The Finnish and Swedish Lapphunds and Lapponian Herders are all descended from the guarding and reindeer herding dogs of Sámi people from northern Scandinavia [25]. The Swedish Lapphund is one of the native Swedish breeds and the other two breeds originate from Finland. Lapphunds are very similar; the biggest difference is the coat color. In Swedish Lapphunds, only black color is accepted whereas in the Finnish Lapphunds all colors are allowed for breeding. Finnish Lapphund and Lapponian Herder were separated in the 1960s on the basis of their coat length [25]. Nowadays these two breeds differ also in overall shape.

Though Pompe disease was described already in the 1960s–1980s in Swedish Lapphunds, its existence in Finnish Lapphunds had not previously been reported by breeders. As far as we know, these Finnish Lapphund dogs are the first correctly diagnosed cases in Finland. Difficulties in diagnosing Pompe disease in Lapphunds might have caused diagnostic delay, but the moderately low mutation frequency in the Finnish Lapphund breed and the pattern of autosomal recessive inheritance appear contributing factors. Our study enables the development of a genetic test for these Scandinavian dog breeds so that this rare but fatal disease can now be eradicated.

Enzyme replacement therapy (ERT) is currently the only effective treatment for human Pompe disease. ERT has been shown to improve ventilator-free survival in patients with infantile-onset Pompe disease. Recent studies indicate that also children and adults benefit from treatment [26], [27]. However, the present form of ERT does not provide a total cure. Therefore, the challenge of improving ERT or introducing alternative therapeutic interventions remains. Given the current promising achievements in the field of gene therapy this approach deserves serious investigation also for treatment of Pompe disease. Being large and well described animal models, the Finnish and Swedish Lapphunds could be instrumental in these new developments.
